# 

**Published:** 1890-06-07

**Authors:** 


					PRlCE ONE PENNY ?_: WITH EXTRA nursing supplement.
TH Mi ssKssaw
"""won in the United Kingdom and Abroad.] *? '?^ Ditto per AnnuiHi 8s.? post free.
isaairtWEs tern Lnfjbmabx '
Sewsii"'
A WEEKLY JOURNAL OF
Smite*, /llbebtctitc, "Murstttg anir H^ijtlantijropg

				

